# Effects of *Moringa oleifera* Leaf Extract on Liver Histopathology: A Systematic Review

**DOI:** 10.1155/2024/6815993

**Published:** 2024-07-04

**Authors:** Titing Nurhayati, Muhamad Farrel Ridho, Putri Teesa Radhiyanti Santoso, Setiawan Setiawan, Hanna Goenawan, Vita Murniati Tarawan

**Affiliations:** ^1^ Department of Biomedical Science Faculty of Medicine Padjadjaran University, Bandung, Indonesia; ^2^ Faculty of Medicine Padjadjaran University, Bandung, Indonesia

## Abstract

**Introduction:**

Moringa leaves (*Moringa oleifera*), which are members of the Moringaceae family, are one of the herbal plants that are widely known in Indonesia. Phytochemical contents of moringa leaf, such as flavonoid, quercetin, and phenolic acid, are believed to have an effect on improvement of NAFLD. Therefore, moringa leaf is considered as one the herbal plants that can be used as supplementation in the form of adjuvant therapy to NAFLD. The study objective of our research is to review the effect of giving moringa leaf to the liver, especially the histopathologic features. This study will be conducted on literature review research design, more specifically in the form of a systematic review. *Research Method*. Five major electronic web databases, including PubMed, Cochrane Library, Google Scholar, Scopus, and ScienceDirect, were used in identifying literature from 2014 to 2023.

**Results:**

From a comprehensive analysis of 13 relevant literature sources, we elucidate the impact of *Moringa oleifera* leaf extract on liver histopathology, glucose, and lipid metabolism. Furthermore, we provide insights into its safety profile concerning human health.

**Conclusion:**

The phytochemical content of *Moringa oleifera* leaf extract had shown a significant benefit in plant medicinal sector. From the research that had been done, *Moringa oleifera* leaf extract contributes to give significant improvement on liver histopathological features, glucose, and lipid metabolism on animal sample model.

## 1. Introduction

Moringa leaves (*Moringa oleifera*), which are members of the Moringaceae family, are one of the herbal plants that are widely known in Indonesia [1, 2]. Every part of the moringa leaf is a storehouse of important nutrients and antinutrients [3–5]. Therefore, moringa leaves are also known as “miracle trees” or magical plants because of their various benefits, including medicinal plants, cosmetics, and food ingredients [1]. Phytochemical contents of moringa leaf, such as flavonoid, quercetin, and phenolic acid, are believed to have an effect on improvement of NAFLD [6–8].

Metabolic syndrome encompasses a group of metabolic irregularities associated with hypertension, central obesity, insulin resistance, and atherogenic dyslipidemia and is closely linked to a heightened likelihood of developing diabetes as well as cardiovascular diseases, both atherogenic and non-atherogenic in nature [9]. One of the important risk factors for metabolic syndrome is lack of physical activity [10]. Several studies have been conducted using accelerometric methods to examine the association between sedentary behaviour and the metabolic syndrome [11]. These studies have shown that increasing the duration and percentage of time spent in sedentary behaviour is associated with increased metabolic risk in adults [11]. One of the concerning manifestations of metabolic syndrome in the liver is non-alcoholic fatty liver disease [12]. Research indicates a robust connection between NAFLD and the characteristics of metabolic syndrome. Insulin resistance serves as a pivotal factor contributing to both NAFLD and metabolic syndrome [13]. Evidence from clinical trials, experimental investigations, and population-based studies suggests that NAFLD could potentially manifest in the liver as a representation of metabolic syndrome [13].

Non-alcoholic fatty liver disease (NAFLD) is identified by liver fat accumulation, or in medical term called steatosis, alongside one of the three conditions: excessive weight or obesity, type 2 diabetes mellitus (T2DM), or being lean or of average weight with indications of metabolic disruption [14]. Non-alcoholic fatty liver disease (NAFLD) is the liver expression of a complex disorder that affects multiple systems [15]. NAFLD as hepatic manifestation of metabolic syndrome is also associated with lack of physical activity as one of the concerning risk factors [16]. Epidemiological studies reveal that NAFLD global prevalence in 2023 is 30% and still increasing in upcoming years [17]. It is considerably associated to increasing in prevalence of sedentary lifestyle in human population [18]. This is supported by the results of a study conducted in South Korea, where there was an increase in the frequency of NAFLD based on the length of time a population had been sitting [19].

Lifestyle modification is one of the most recommended ways for patients with NAFLD or to prevent NAFLD itself. Available evidence suggests that as little as 3% to 5% weight loss is needed to improve simple hepatic steatosis [20–23]. In addition, pharmacological therapy can also be given to individuals who have NAFLD [24]. The goals for pharmacological therapy of NAFLD should target the fat accumulation that occurs and the injury and fibrosis that are consequences of NAFLD [25]. Another way that can be done as a management or prevention of NAFLD is supplementation in the form of adjuvants. Many adjuvants are available in the market, one of which is herbal plants. Herbal plants that are believed to have an effect on NAFLD are *Moringa oleifera* leaves [26].

Until now, research and clinical trials on moringa leaves have begun to develop and are being intensively carried out. However, knowledge about the utilization of moringa leaf in Indonesia is still low [27]. Therefore, we are interested in conducting this review on the effect of giving moringa leaf to the liver. This study will be conducted on literature review research design, more specifically in the form of a systematic review. This review was done to see the effect of the nutrient content and active substances in moringa leaf on tissue structure and liver function.

## 2. Research Method

This study is conducted on literature review research design, more specifically in the form of systematic review. This review identifies the effects of *Moringa oleifera* leaf extract on liver structure and function in animal sample model. We used the PICO method approach to generate the clinical question for designing this study. The population/patient we use for this study is NAFLD patient. We use *Moringa oleifera* leaf extract as the intervention to the population/patient. The intervention was compared with standard diet control group. Lastly, we use liver histopathology as the outcome analysis.

Five major web electronic databases, including PubMed, Scopus, Google Scholar, Cochrane, and ScienceDirect, were used for identifying the literature that provides explanation on effects of *Moringa oleifera* leaf extract on liver histopathology. Search of the literature was conducted through a thorough searching process by using some concepts as *Moringa oleifera*, leaf extract, liver histopathology, rat, mice, and guinea pigs. Every concept was searched and combined using Boolean Operators “AND.” Other similar terms of the concepts also were searched and combined using Boolean Operators “OR.” Research conducted from 2014 to 2023 was encompassed in the analysis. The pertinent research papers were gathered and will be selected through thorough selection process. Before the identified research papers were selected, the research articles identified as duplicates were eliminated.

The selection process is conducted in two steps. The initial step involved screening the titles and abstracts of the articles and studies identified from the search results. The title and abstract were screened by matching the criteria of the inclusion. Inclusion criteria used in the selection process were papers that explained the effects of *Moringa oleifera* on liver histopathology. Also, we only included experimental studies that used rat, mice, or guinea pig as the animal sample model. Other research designs, such as review, comment, and letter, were excluded. Experimental studies that used animal sample model besides rat, mice, or guinea pig were also excluded. We also verified that the chosen articles and studies were indexed in the Scimago Journal and Country Rank (SJR) and Science and Technology Index (SINTA) for journal publications. Two reviewers (MFR and TN) independently screened the titles and abstracts in duplicate with >80% agreement. In cases where screening results were conflicting, a third reviewer (PT) was involved to resolve the discrepancies.

The next selection steps were to screen the full text of the articles that passed the first selection process. In the full-text screening, we carefully reviewed the articles to meet the right criteria and outcome related to the effects of *Moringa oleifera* leaf extract on liver histopathology. The same two reviewers (MFR and TH) did the full-text screening independently and in duplicate with >80% agreement. The same third reviewer (PT) also did take part to resolve some conflicts.

To assess the quality of the literature that included, we used Joanna Briggs Institute (JBI) Critical Appraisal Checklist [28]. The quasi-experimental checklist type was used to evaluate the quality of the literature [28]. The studies were considered of good quality if they obtained a minimum of 60% “YES” responses across all questions and of lower quality if they did not meet this threshold. The critical appraisal was done by the same two reviewers (MFR and TH) independently and in duplicate.

Two reviewers (MFR and TH) extracted data independently and in duplicate using a standardized form designed for this study. Several data provided by the articles that were eligible were collected. We retrieved the following data: research objective, study design, target groups, and major findings. The data were compiled using Microsoft Excel and arranged in the form of table.

Various varieties of *Moringa oleifera* leaf extracts, model experiments, phytochemical content, and outcomes were explained and compared. Besides liver histopathology, we also explain the effects of *Moringa oleifera* leaf extract on glucose and lipid metabolism. In addition, we also provide explanation on safety of *Moringa oleifera* leaf extract usage, including lethal dosage and toxicological properties.

## 3. Result

### 3.1. Studies Included in the Review

Six hundred twenty-seven studies were identified through literature search in 5 databases. We found 0 studies on PubMed, 0 studies on Cochrane Library, 70 studies on Scopus, 26 studies on ScienceDirect, and 531 studies on Google Scholar. Before screening, we deleted 129 studies identified as duplicate. After removal of duplicate studies, we screened the title and abstract of 498 studies by matching the inclusion and exclusion criteria that we used. Then, 435 studies were excluded and 63 studies remained. Studies that passed the first selection then went through the full-text screening. After that, 50 studies were excluded and 13 studies remained. These 13 studies have met our inclusion criteria, so we used these included studies for this literature review research.

### 3.2. Data Collected from the Included Studies

All included studies are experimental studies, specifically the included literatures are a randomized control trial in animal sample model. All studies explained the effects of *Moringa oleifera leaf extract* on liver histopathology. The principle findings and all collected data from the included literature were summarized in Table 1.

The included studies were conducted by experts from Indonesia, Mexico, Egypt, Nigeria, Iran, Saudi Arabia, South Korea, Pakistan, and Libya.

**Figure 1:**
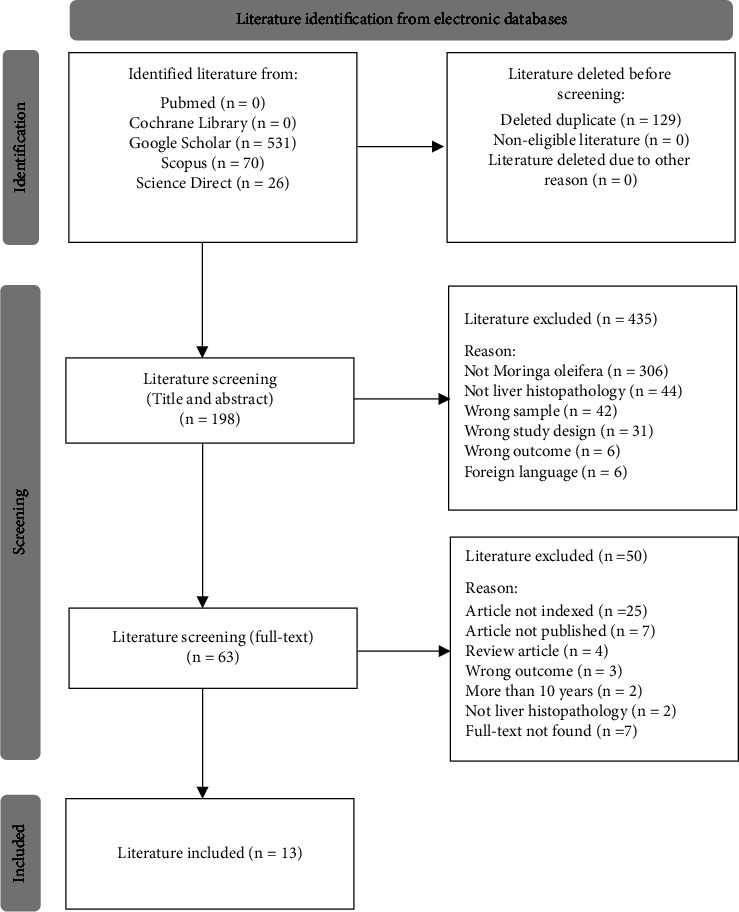
Flow diagram of literature selection process from the electronic databases [50].

In addition, some references, besides included literature, were chosen to provide the effects of *Moringa oleifera* leaf extract on glucose and lipid metabolism. Glucose and lipid metabolism were also illustrated due to their involvement in the pathogenesis and pathophysiology of liver disease, specifically non-alcoholic fatty liver disease. The phytochemical contents of *Moringa oleifera* are provided in the form of Table 2. In addition, we also provide the safety of *Moringa oleifera* leaf extract usage by explaining its lethal dosage.  Figure 1 shows the flow diagram of literature selection process from the electronic databases.

## 4. Discussion

### 4.1. Phytochemical Content of *Moringa oleifera* Leaf Extract

The phytochemical composition of *Moringa oleifera* leaf primarily comprises phenolic compounds, which are essential plant-derived micronutrients [1]. Phenolic compounds are a group of compounds characterized by hydroxyl groups directly attached to aromatic structures, encompassing phenolic acids, flavonoids, xanthones, quinones, coumarins, tannins, and lignans [2]. Phenolic compounds play significant roles in safeguarding plants against physical damage, ultraviolet radiation, oxidative stress, and similar factors. Currently, there is growing interest in uncovering, extracting, and enhancing phytochemicals due to their potential to serve as alternatives to synthetic drugs with fewer side effects. Unquestionably, dried *Moringa oleifera* leaves serve as significant natural reservoirs of phenolic compounds [3].

This finding confirmed the phenolic compound stated in the included literature. Some of the included literature also conducted an analysis on phytochemical component in the *Moringa oleifera.* The analysis was conducted to reassuring the presence of the phytochemical compound in the *Moringa oleifera* leaf extract. Most of the articles that conducted analysis on phytochemical content stated that they identified phenolic acid and quercetin as the key findings for the phytochemical content of *Moringa oleifera* leaf extract [29–41].

### 4.2. Effects of *Moringa oleifera* Leaf Extract on Glucose Metabolism

As previously noted, a variety of polyphenols are present in *Moringa oleifera*. Many compounds contained in moringa leaf are proven to have involvement in the metabolism of glucose homeostasis [46]. Among the most noteworthy are flavonoids like quercetin and kaempferol, as well as phenolic acids such as chlorogenic acid and caffeoylquinic acid [51]. These compounds appear to possess antihyperglycemic attributes, acting as competitive inhibitors of sodium-glucose linked transporter type 1 (SGLT1) in the mucosa of the small intestine (specifically, the duodenum and jejunum). This action leads to a reduction in the absorption of glucose in the intestines [52]. However, it is important to note that glucose absorption involves other mechanisms, including the involvement of glucose transporter 2 (GLUT2), which can be influenced by the presence of circulating glucose, directing it towards the basolateral membrane of the small intestine [53].

*Moringa oleifera* has been investigated for its potential in managing glucose metabolism by contributing to the reduction of glucose levels. One suggested mechanism involves quercetin, which can function as an inhibitor of GLUT2 at the apical surface [54]. It is important to note that this influence is specific to GLUT2 and does not affect GLUT5 or SGLT1 [55]. Moreover, quercetin has demonstrated the ability to activate adenosine monophosphate-activated protein kinase (AMPK), thereby enhancing glucose uptake through the stimulation of GLUT4 in skeletal muscle. Additionally, it plays a role in diminishing glucose production by suppressing the activity of phosphoenolpyruvate carboxykinase (PEPCK) and glucose-6-phosphatase (G6Pase) in the liver [56].

The aqueous leaf extract of *Moringa oleifera* has demonstrated the ability to hinder the activity of *α*-glucosidase, pancreatic *α*-amylase, and intestinal sucrose, contributing to its antihyperglycemic effects [57]. These inhibitory outcomes are attributed to the presence of phenols, flavonoids, and tannins in *Moringa oleifera*. By delaying the digestion of carbohydrates through enzyme inhibition, there is a consequent reduction in post-meal hyperglycemia and levels of hemoglobin A1C (HbA1C). The inhibitory impact of flavonoids like quercetin and kaempferol can be explained biochemically by their increased number of hydroxyl groups on the B ring and the presence of a 2,3-double bond [58]. Moreover, these compounds have undergone scrutiny for their protective and regenerative effects on pancreatic beta-cells, leading to enhanced insulin production and release [59]. Quercetin, for instance, stimulates insulin secretion by activating the extracellular signal-regulated kinase 1/2 (ERK1/2) pathway and simultaneously shields pancreatic beta-cells against oxidative harm [5].

This mechanism of action of polyphenols contained in *Moringa oleifera* leaf extract is in parallel with the findings of the included literature. Some studies showed that there is significant reduction of blood glucose level and improvement of insulin sensitivity measured in group of animals treated by *Moringa oleifera* leaf extract [29, 33, 36, 37, 49]. From the studies included, we conclude that dosage of *Moringa oleifera* leaf extract of more than 200 mg/kg body weight showed more significant result of glucose and insulin profile than the lower dosage [29, 33, 36, 37, 49].

### 4.3. Effects of *Moringa oleifera* Leaf Extract on Lipid Metabolism

Phenolic compounds, as well as flavonoids, have an important role in lipid regulation [49, 60]. Therefore, *Moringa oleifera* has also been considered as a potential hypolipidemic agent. An aqueous extract derived from *Moringa oleifera* leaf has been reported to possess lipid-lowering attributes by diminishing the creation of cholesterol micelles and inhibiting enzymes like pancreatic lipase and pancreatic cholesterol esterase, as well as impeding bile acid binding [57]. Furthermore, an increase in the expression of peroxisome proliferator-activated receptors (PPARs) *α*1 (PPAR*α*1) and *γ* (PPAR*γ*) has been observed in rats administered *Moringa oleifera* seed powder [61]. PPARs play a pivotal role in lipid metabolism, ketogenesis, and cellular energy balance, and they are present in various organs, including the liver, brain, muscles, and heart, among others. Additionally, PPARs are implicated in processes such as inflammation, immunity, and glucose regulation [62]. Another research demonstrated that administering *Moringa oleifera* leaf extract during the process of adipogenic differentiation leads to a decrease in inflammation and lipid buildup, while also triggering thermogenesis through the activation of key proteins such as uncoupling protein 1 (UCP1), sirtuin 1 (SIRT1), peroxisome proliferator-activated receptor alpha (PPAR*α*), and coactivator 1 alpha (PGC1*α*). Furthermore, *Moringa oleifera* Lam. induces the expression of heme oxygenase-1 (HO-1), a well-recognized protective and antioxidant enzyme [63].

Those findings on hypolipidemic effects of *Moringa oleifera* are in parallel with the outcomes of the included literature. Most of the studies provide explanation of the resulting effects of *Moringa oleifera* leaf extract on lipid profile. The collected data of included literatureexplained that *Moringa oleifera* leaf extract provided significant reduction of triglyceride, LDL-C, VLDL-C, and cholesterol in group fed by *Moringa oleifera* leaf extract [29, 33, 35, 36, 49]. Besides that, included literature also stated that there is an improvement of HDL-C in lipid profile of group fed by *Moringa oleifera* leaf extract [36].

By now, it is recognized that the consumption of a high-fat diet stimulates the generation of proinflammatory cytokines like IL1B and TNFA, which are activated via the NFkB pathway [64, 65]. This dietary pattern also results in an elevation of mitochondrial ROS production. These free radicals contribute to the peroxidation of lipids on cellular and organelle membranes, leading to the formation of malondialdehyde (MDA), which serves as an indicative marker of oxidative stress [66]. Choi and Das illustrated an increase in lipid peroxidation products such as MDA and 4HNE in a model of NAFLD, attributable to the generation of reactive oxygen species [67, 68]. Some studies revealed that the application of moringa results in a decline in the generation of MDA, attributed to a reduction in ROS production [49]. This finding is in parallel with some included studies that also show significant reduction of lipid peroxidation in liver characterized by reduction of MDA level in group fed by *Moringa oleifera* leaf extract [29, 30, 34, 38].

From the studies included, we conclude that dosage of *Moringa oleifera* leaf extract of more than 200 mg/kg body weight showed more significant result on lipid profile and MDA level than the lower dosage [29, 30, 34, 38].

### 4.4. Effects of *Moringa oleifera* Leaf Extract on Liver Histopathology

Several hypotheses have been proposed to explain the mechanisms of liver injury leading to liver steatosis. These include liver injury caused by hepatotoxins, damage from a diet high in fat, and liver injury related to metabolic disorders [69–71]. All those etiologies may trigger liver damage by means of oxidative stress, inflammation, fibrogenesis, and liver necrosis. Sudden damage to liver cells disrupts their transport function and the permeability of their membranes, causing the release of marker enzymes [72]. The internal processes of damage within hepatocytes involve the creation of reactive metabolites, reduction of glutathione levels, and protein alkylation, particularly affecting mitochondrial proteins [73]. These initial actions prompt the opening of the mitochondrial membrane, resulting in permeability transition. The deterioration of the mitochondrial membrane permeability transition occurs prior to the failure of the cell's outer membrane, leading to cell swelling and the release of cellular contents. This ultimately culminates in cell death through a process known as oncotic necrosis [74].

The evaluation of liver damage was gauged through the measurement of serum levels of ALT and AST, which are widely recognized as prevalent indicators of liver harm. When the integrity of the liver cell membrane is compromised, several enzymes that typically reside within the cell's cytosol are discharged into the bloodstream. The concentration of these enzymes in the serum provides a valuable quantitative gauge of the magnitude and nature of hepatocellular injury [71].

Liver injury, in the term of NAFLD, is often characterized by macrovesicular steatosis, ballooning degeneration of hepatocytes, scattered (mainly lobular) inflammation and apoptotic bodies, and Mallory-Denk bodies (MDBs). Fibrosis also often presents alongside the other histological features [75].

The included literature had revealed that there is significant improvement in the histopathological analysis of the liver tissue conducted on animal sample treated by *Moringa oleifera* leaf extract. All studies included explained that after administration of *Moringa oleifera* leaf extract, there is significant reduction of fibrosis, hepatic cells necrosis, lipid accumulation, inflammatory cells infiltration, hepatocellular degeneration, vesicular congestion, and sinusoidal dilatation. Various dosages of *Moringa oleifera* leaf extract had been analyzed. Higher dosage of *Moringa oleifera* extract showed more reduction on the histopathological feature of the liver tissue, while lower dosage did not give any notable improvement on the histopathological feature of the liver tissue [29–41, 49]. Some studies included also measured liver injury biomarkers as other findings besides liver histopathological features. These studies stated that there is significant reduction on the AST, ALT, ALP, SGOT, and SGPT in the group of animals treated by *Moringa oleifera* leaf extract [29–31, 33–36, 40, 41, 49].

From the studies included, we conclude that dosage of *Moringa oleifera* leaf extract of more than 200 mg/kg body weight showed more significant result on liver histopathological feature and liver biomarkers than the lower dosage [29–41].

### 4.5. Safety of *Moringa oleifera* Leaf Extract

Earlier investigations were carried out on animal models to analyze the oral toxicity (LD50) of *Moringa oleifera* leaf extract [76]. The findings indicated that the aqueous leaf extract of *Moringa oleifera* did not result in any fatalities even at the highest administered dose of 6400 mg/kg body weight [77]. A study conducted by Fouad et al. [78] on acute oral toxicity reported that *Moringa oleifera* leaf extract demonstrated non-lethal effects on animals at a dose of 2000 mg/kg body weight and mentioned that animals may display some adverse changes at doses beyond this level. Moreover, Diallo et al. [79] documented that the aqueous leaf extract of *Moringa oleifera* was safe even at dosages as high as 5000 mg/kg body weight.

These findings suggest that the aqueous leaf extract of *Moringa oleifera* is safe for oral consumption, displaying no lethal effects during acute administration. It is noteworthy that a dose of 2 g/kg body weight was identified as the threshold for medicinal plant toxicity in acute oral toxicity studies [80]. However, this safety assertion might not hold true for medicinal plants consumed over an extended period. Slight lethargy was observed in animals receiving doses above 1600 mg/kg body weight during acute administration, which aligns with the observations of Adedapo et al., who noted toxic changes in animals above 2000 mg/kg body weight. The LD50 from an acute oral intraperitoneal toxicity study for *Moringa oleifera* leaf extract was determined to be 1585 mg/kg body weight.

Previous study conducted by Kushwaha et al. [81] documented the adverse outcomes of *Moringa oleifera* leaf extract in studies involving human subjects. In this research, a group of 30 post-menopausal women was provided a daily supplementation of 7 g of *Moringa oleifera* leaf powder for a duration of 12 weeks, and their results were compared with those of a control group. The study revealed a rise in antioxidant biomarkers that contributed to hypoglycemic and hypolipidemic effects, all without inducing any harmful effects.

## 5. Conclusion

The phytochemical content of *Moringa oleifera* leaf extract had shown a significant benefit in plant medicinal sector. From the research that had been done, *Moringa oleifera* leaf extract contributes to give significant improvement on liver histopathological features, glucose, and lipid metabolism on animal sample model. In the coming times, further investigation into this medicinal plant could offer promising prospects regarding its effectiveness and safety as a therapeutic solution for NAFLD.

### 5.1. Limitation

Due to the various types of extracts employed in studies, it becomes imperative to determine if specific phytochemicals within *Moringa oleifera* leaf extract are present in each variant. Consequently, additional investigations are warranted to discern the most potent type of *Moringa oleifera* leaf extract and its optimal bioavailability.

## Figures and Tables

**Table 1:** Studies dealing with the effects of *Moringa oleifera* leaf extract on liver histopathology [29–41].

Research objective	Study design	Target groups	Major findings	Other findings	References
To investigate how a water-based extract derived from *Moringa oleifera* affects liver microRNAs, gene and protein expression, and histological and biochemical factors, within an experimental NASH model, with a focus on its potential hepatoprotective properties	RCT	Classification of C57BL/6J strain mice:	(a) Significant diminution and reduction of inflammatory nodules, extracellular matrix quantification, collagens, presence of *α*SMA, and activation of hepatic stellate cells measured by histological analysis of liver in MO treated group	(a) Significant weight reduction of animal body weight, liver weight, and epididymal fat pad weight in MO treated group	[29]
		(1) Standard diet control group (*n* = 5)		(b) Significant reduction of serum glucose level due to preserved insulin sensitivity in MO treated group	
		(2) High-fat diet group (*n* = 10) (a) High-fat diet group without treatment (*n* = 5) (b) High-fat diet group supplied by 150 *μ*L aqueous *Moringa oleifera* extract (62.5 mg/mL) (*n* = 5)		(c) Showed reduction of AST, ALT, cholesterol, insulin, resistin, leptin, and PAI-1 levels in MO treated group	
				(d) Significant reduction of lipid peroxidation due to lower MDA levels in MO treated group	

To investigate the impacts of a nanoplatform containing *Moringa oleifera* extract, along with vitamin C and selenium (MO/asc.-Se-NPs), on the prevention and treatment of hepatocellular carcinoma (HCC)	RCT	Classification of male Wistar albino rats:	(a) Significant reduction of perivascular infiltration with fibroblast, vessel congestion, and lymphocyte cell infiltration measured by histological analysis of liver in MO prevention and MO treated groups	(a) Showed reduction of AST, ALT, and albumin in MO prevention and MO treatment groups	[30]
		(1) Control normal group (*n* = 5)		(b) Significant reduction of lipid peroxidation due to lower MDA level in MO prevention and MO treated groups	
		(2) MO/asc.-Se-NPs supplement group (1 mL/kg weekly) (*n* = 5)			
		(3) HCC induction group			
		(4) HCC induction + MO/asc.-Se-NPs (1 mL/kg weekly) (*n* = 5)			
		(5) MO/asc.-Se-NPs treatment (1 mL/kg weekly) after HCC induction (*n* = 5)			

To investigate the potential hepatoprotective and antioxidant effects of an ethanol extract of *Moringa oleifera* (EEMO) against hepatocellular injury and oxidative stress induced by potassium dichromate (K2Cr2O7) in male Wistar rats	RCT	Classification of male Swiss albino rat:	(a) Showed amelioration of hepatocellular degeneration and severe necrosis measured by histological analysis of liver in EEMO treated group	(a) Significant reduction of final body weight, liver weight, percentage body weight change, and relative liver weight in EEMO treated group (b) Significant reduction of AST and ALT in EEMO treated group (c) Significant improvement of MDA level, SOD activity, and GST activity in EEMO treated group	[34]
		(1) Negative control group (*n* = 5)			
		(2) Positive control group fed by K2Cr2O712 mg/kg body weight weekly (*n* = 5)			
		(3) Rats fed by EEMO 3.5 mg/kg body weight weekly (*n* = 5)			
		(4) Rats fed by EEMO 7 mg/kg body weight weekly (*n* = 5)			
		(5) Rats fed by K2Cr2O712 mg/kg body weight + EEMO 3.5 mg/kg body weight weekly (*n* = 5)			
		(6) Rats fed by K2Cr2O7 12 mg/kg body weight + EEMO 7 mg/kg body weight weekly (*n* = 5)			

To investigate the effects of medicinal properties like quercetin, gallic acid, and caffeic acid of stem and leaf extract of *Moringa oleifera* in protecting liver steatosis in high-fat diet fed rats	RCT	Classification of male Wistar rat:	(a) Significant reduction of macro- and microvesicular fat, hepatocyte swelling measured by histological analysis of liver in MO treated group	(a) Showed reduction of body weight and visceral fat weight in MO treated group (b) Significant reduction of AST and ALT level in MO treated group (c) Significant reduction of TG, TC, LDL-C, and VLDL-C in MO treated group	[35]
		(1) Standard diet control group (*n* = 5)			
		(2) High-fat diet group (*n* = 5)			
		(3) High-fat diet + MO leaf powder 2% (*n* = 5)			
		(4) High-fat diet + MO leaf powder 4% (*n* = 5)			
		(5) High-fat diet + MO stem powder 2% (*n* = 5)			
		(6) High-fat diet + MO stem powder 4% (*n* = 5)			

To investigate the effects of *Moringa oleifera* leaf extract on lead acetate poisoning	RCT	Classification of mice:	(a) Significant reduction of cytoplasmic vacuolation, granulation in some cells, pyknosis of the nuclei, inflammatory cell infiltration, vessel congestion, and hepatocyte necrosis measured by histological analysis of liver in MO treated group	(a) Significant improvement of AST, ALT, and ALP in MO treated group (b) Significant improvement of cholesterol, HDL-C, LDL-C, and TG in MO treated group (c) Significant reduction of serum glucose level in MO treated group	[36]
		(1) Normal control group (*n* = 15)			
		(2) Mice fed by MO leaf extract 300 mg/kg body weight weekly (*n* = 15)			
		(3) Mice fed by 1/10LD50 of Pb (C2H3O2)2 (60 mg/kg body weight) for 45 days (*n* = 15)			
		(4) Mice fed by 1/10 LD50 of Pb (C2H3O2)2 (60 mg/kg body weight) + by MO leaf extract 300 mg/kg body weight for 45 days (*n* = 15)			

To study the effectiveness of fermented extract from *Moringa oleifera* (FM) in addressing glucose intolerance and the buildup of liver fats induced by a high-fat diet (HFD)	RCT	Classification of C57BL/6J rat:	(a) Significant reduction of hepatic lipid accumulation in MO treated group measured by histological analysis of liver	(a) Significant better glucose tolerance in MO treated group	[37]
		(1) Normal control group			
		(2) Rat fed by high-fat diet group			
		(3) Rat fed by high-fat diet + fermented MO extract 250 mg/kg body weight daily			
		(4) Rat fed by high-fat diet + non-fermented MO extract 250 mg/kg body weight daily			

To investigate the protective effect of *Moringa oleifera* leaf extract on liver and kidney damage induced by gentamicin in rats	RCT	Classification of rat:	(a) Significant reduction of hepatocyte degeneration, sinusoid dilatation, and hepatic cell necrosis in MO treated group measured by histological analysis of liver	(a) Significant reduction of lipid peroxidation characterized by lowered MDA level in MO treated group	[38]
		(1) Normal control group (*n* = 5)			
		(2) Rat fed by gentamicin 80 mg/kg body weight (*n* = 5)			
		(3) Rat fed by gentamicin 80 mg/kg body weight + MO leaf extract 150 mg/kg body weight (*n* = 5)			
		(4) Rat fed by gentamicin 80 mg/kg body weight + MO leaf extract 300 mg/kg body weight (*n* = 5)			
		(5) Rat fed by gentamicin 80 mg/kg body weight + MO leaf extract 300 mg/kg body weight (*n* = 5)			

To investigate the effects of multilevel doses of *Moringa oleifera* leaf extract on the microscopic appearance of the livers of formalin-induced Wistar rats	RCT	Classification of male Wistar rat:	(a) Significant reduction of hepatocyte degeneration and necrosis measured by histological analysis of liver tissue using Manja Roenigk scoring in MO treated group	—	[39]
		(1) Negative control group (*n* = 5)			
		(2) formalin 100 mg/kg body weight daily (*n* = 5)			
		(3) Rat fed by normal diet + MO leaf extract 200 mg/kg body weight daily + formalin 100 mg/kg body weight daily			
		(4) Rat fed by normal diet + MO leaf extract 400 mg/kg body weight daily + formalin 100 mg/kg body weight daily			
		(5) Rat fed by normal diet + MO leaf extract 800 mg/kg body weight daily + formalin 100 mg/kg body weight daily			

To investigate the effect and effective dose of the ethyl acetate fraction of moringa leaf on histopathological features of the liver and SGPT and SGOT levels in monosodium glutamate-induced rats	RCT	Classification for rat:	(a) Significant reduction of hepatocyte degeneration and lipid accumulation between hepatic cells in MO treated group measured by histological analysis of liver	(a) Significant reduction of SGOT and SGPT levels in MO treated group	[40]
		(1) Normal control group (*n* = 5)			
		(2) Rat fed by MSG 3.6 mg/g body weight (*n* = 5)			
		(3) Rat fed by MSG 3.6 mg/g body weight + MO ethyl acetate fraction 20.17 mg/g body weight (*n* = 5)			
		(4) Rat fed by MSG 3.6 mg/g body weight + MO ethyl acetate fraction 30.26 mg/g body weight (*n* = 5)			
		(5) Rat fed by MSG 3.6 mg/g body weight + MO ethyl acetate fraction 45.39 mg/g body weight (*n* = 5)			

To investigate the healing capabilities of *Moringa oleifera* in the context of fatty liver induced by ethanol consumption	RCT	Classification of male mice:	(a) Significant reduction of hepatic steatosis, lobular inflammation, and hepatic ballooning in MO treated group measured by histological analysis of liver	(a) Significant reduction of ALT, AST, and TG in MO treated group (b) Significant reduction of TNF-*α* in MO treated group measured by immunohistochemistry analysis of liver	[41]
		(1) Normal control group (*n* = 8)			
		(2) Mice fed by 30% ethanol 100 *μ*l (*n* = 8) daily			
		(3) Mice fed by 30% ethanol 100 *μ*l + MO extract 100 mg/kg body weight (*n* = 8)			
		(4) Mice fed by 30% ethanol 100 *μ*l + MO extract 200 mg/kg body weight (*n* = 8)			
		(5) Mice fed by 30% ethanol 100 *μ*l + MO extract 400 mg/kg body weight (*n* = 8)			

To investigate the phytochemical capacity of *Moringa oleifera* in countering oxidative stress induced by acetaminophen and its role in influencing liver biomarkers, liver tissue structure, and the JNK pathway within the MAPK signaling cascade	RCT	Classification of Wistar rat:	(a) Significant improvement of hepatocyte degeneration, hepatic cell necrosis, inflammation, and vascular injury in MO treated group measured by histological analysis of liver	(a) Significant reduction of ALT, ALP, AST, bilirubin, and GGT levels in MO treated group	[31]
		(1) Normal control group (*n* = 10)			
		(2) Rat fed by acetaminophen 200 mg/kg body weight (*n* = 10)			
		(3) Rat fed by acetaminophen 200 mg/kg body weight + silymarin 200 mg/kg body weight (*n* = 10)			
		Rat fed by acetaminophen 200 mg/kg body weight MO extract (200 mg/kg body weight) (*n* = 10)			

To investigate the impacts of *Moringa oleifera* leaf extract on alterations caused by bisphenol-A (changes in hepatocyte diameter and vacuolization) in the livers of rats	RCT	Classification of albino rat:	(a) Significant reduction of hepatocyte diameter and hepatic vacuolization in MO treated group measured by histological analysis of liver		[32]
		(1) Normal control group (*n* = 8)			
		(2) Rat fed by BPA 50 mg/kg body weight (*n* = 8)			
		(3) Rat fed by BPA 50 mg/kg body weight + MO leaf extract 250 mg/kg body weight (*n* = 8)			
		(4) Rat fed by BPA 50 mg/kg body weight + MO leaf extract 500 mg/kg body weight (*n* = 8)			

To investigate the potential of antidiabetic impact of methanolic extracts from *Moringa oleifera* leaf, seeds, and a combination of both, administered at a dosage of 500 mg/kg of body weight per day	RCT	Classification of mice:	(a) Showed reduction of accumulation of fat vacuoles in hepatocytes, inflammatory cell infiltration, vascular dilatation and congestion, and fibrosis at the pericellular and perisinusoidal level in MO treated group measured by histological analysis of liver	(a) Significant reduction of blood glucose level in MO treated group (b) Significant reduction of ALT, AST, and ALP activity in MO treated group (c) Significant reduction of total cholesterol and triglycerides in MO treated group	[33]
		(1) Normal control group (*n* = 3)			
		(2) Alloxan induced diabetic mice group (*n* = 3)			
		(3) Diabetic mice + insulin administration 0.7 mg/kg body weight daily for 1 month (*n* = 3)			
		(4) Diabetic mice + MO leaf extract 500 mg/kg body weight daily for 1 month (*n* = 3)			
		(5) Diabetic mice + MO seed extract 500 mg/kg body weight daily for 1 month (*n* = 3)			
		(6) Diabetic mice + MO combination extract 500 mg/kg body weight daily for 1 month (*n* = 3)			
		(7) Diabetic mice + MO leaf extract 500 mg/kg body weight daily for 3 months (*n* = 3)			
		(8) Diabetic mice + MO seed extract 500 mg/kg body weight daily for 3 months (*n* = 3)			
		(9) Diabetic mice + MO combination extract 500 mg/kg body weight daily for 3 month (*n* = 3)			

RCT: randomized control trial; microRNA: microribonucleic acid; NASH: non-alcoholic steatohepatitis; MO: *Moringa oleifera*; AST: aspartate aminotransferase; ALT: alanine transaminase; PAI-1: plasminogen activator inhibitor-1; *α*SMA: alpha-smooth muscle actin; MDA: malondialdehyde; ASC: ascorbic acid; -Se-: selenium; NPs: nanoplatform; mL: milliliter; mg: milligram; *n*: natural number; *μ*L: microliter; kg: kilogram; HCC: hepatocellular carcinoma; EEMO: ethanol extract of *Moringa oleifera*; K2Cr2O7: potassium dichromate; SOD: superoxide dismutase; GST: glutathione S-transferase; TG: triglyceride; TC: total cholesterol; LDL-C: low density lipoprotein-cholesterol; VLDL-C: very low density lipoprotein-cholesterol; LD50: lethal dose, 50%; Pb (C2H3O2)2: lead (II) acetate; HDL-C: high density lipoprotein-cholesterol; HFD: high-fat diet; FM: fermented extract of *Moringa oleifera*; SGPT: serum glutamic pyruvic transaminase; SGOT: serum glutamic oxaloacetic transaminase; MSG: monosodium glutamate; TNF-*α*: tumor necrosis factor-*α*; JNK: c-Jun N-terminal kinase; MAPK: mitogen-activated protein kinases; GGT: gamma-glutamyltransferase; BPA: bisphenol-A; ALP: alkaline phosphatase.

**Table 2:** Phenolic compounds of *Moringa oleifera* leaf extract [42–49].

Constituent	Content in leaf (mg/g)	Extraction method
*Phenolic acid*		
Caffeic acid	6.28	Distilled water
Chlorogenic acid	50.69	Distilled water
	79.31	Distilled water
Ellagic acid	33.15	Distilled water
	52.95	Distilled water
Gallic acid	32.45	Distilled water
	105.67	Distilled water
	1.03	Distilled water
Salicylic acid	0.24	Ethanol extract
Ferulic acid	0.128	Distilled water

*Flavonoid*		
Isoquercitrin	64.53	Distilled water
	75.65	Distilled water
Quercitrin	29.74	Distilled water
	74.90	Distilled water
Epicatechin	10.03	Distilled water
	29.73	Distilled water
Catechin	15.27	Distilled water
	20.19	Distilled water
Rutin	15.27	Distilled water
	60.38	Distilled water
Quercetin	137.81	Methanol 70%
	10.7	Distilled water
	47.91	Pressurized hot water extraction
	38.2	Distilled water
Kaempferol	18.23	Distilled water
	106.75	Distilled water
	47.4	Pressurized hot water extraction
	2.8	Methanol 70%
Myricetin	1.53	Methanol 80%
Vanillin	0.137	Distilled water

## Data Availability

All relevant data are included within the article and the attached supplementary information file.

## Supplementary Materials

Supplementary Materials Table presenting the results of the quality assessment, providing detailed information on the quality indicators of the included literature using JBI quasi-experimental critical appraisal tools [28].
