# Supplementary material for: Effects of Moringa oleifera Leaf Extract on Liver Histopathology: A Systematic Review
Source: J Nutr Metab. 2024 Jul 4;2024:6815993. doi: 10.1155/2024/6815993 (PMC11239234; doi:10.1155/2024/6815993)
Supplement: Supplementary Materials — Table presenting the results of the quality assessment, providing detailed information on the quality indicators of the included literature using JBI quasi-experimental critical appraisal tools [28]. [file 6815993.f1.docx]

**Supplementary Table 1. Quality Assessment**

| Author (Publication Year) | Quality Assessment Questions | | | | | | | | | |
| --- | --- | --- | --- | --- | --- | --- | --- | --- | --- | --- |
|  | Is it clear in the study what is the ‘cause’ and what is the ‘effect’ | Were the participants included in any comparisons similar? | Were the participants included in any comparisons receiving similar treatment/care, other than the exposure or intervention of interest? | Was there a control group? | Were there multiple measurements of the outcome both pre and post the intervention/exposure? | Was follow up complete and if not, were differences between groups in terms of their follow up adequately described and analyzed? | Were the outcomes of participants included in any comparisons measured in the same way? | Were outcomes measured in reliable way? | Was appropriate statistical analysis used? | Overall appraisal |
| Monraz-Mendez *et al.* (2022) | Yes | Yes | Yes | Yes | Yes | Yes | Yes | Yes | Yes | Include |
| Ebrahem *et al.* (2022) | Yes | Yes | Yes | Yes | Yes | Yes | Yes | Yes | Yes | Include |
| Akinwumi Kazeem *et al.* (2019) | Yes | Yes | Yes | Yes | Yes | Yes | Yes | Yes | Yes | Include |
| A. Asgari-Kafrani *et al.* (2019) | Yes | Yes | Yes | Yes | Yes | Yes | Yes | Yes | Yes | Include |
| S.J. Melebary and Moustafa H.R. Elnaggar (2022) | Yes | Yes | Yes | Yes | Yes | Yes | Yes | Yes | Yes | Include |
| Hyunchae Joung *et al.* (2017) | Yes | Yes | Yes | Yes | Yes | Yes | Yes | Yes | Yes | Include |
| H.N. Wijayanti *et al.* (2022) | Yes | Yes | Yes | Yes | Yes | Yes | Yes | Yes | Yes | Include |
| Okta Hardianti Putri *et al.* (2018) | Yes | Yes | Yes | Yes | Yes | Yes | Yes | Yes | Yes | Include |
| A.A. Hesti Wulan S *et al.* (2019) | Yes | Yes | Yes | Yes | Yes | Yes | Yes | Yes | Yes | Include |
| Chang Geon Kim *et al.* (2022) | Yes | Yes | Yes | Yes | Yes | Yes | Yes | Yes | Yes | Include |
| Noor Younis *et al.* (2022) | Yes | Yes | Yes | Yes | Yes | Yes | Yes | Yes | Yes | Include |
| Attya Zaheer *et al.* (2020) | Yes | Yes | Yes | Yes | Yes | Yes | Yes | Yes | Yes | Include |
| Badriyah Aljazzaf *et al.* (2023) | Yes | Yes | Yes | Yes | Yes | Yes | Yes | Yes | Yes | Include |
